# Nutrient-Wide Association Study for Dementia Risks: A Prospective Cohort Study in Middle-Aged and Older Adults

**DOI:** 10.3390/nu17121960

**Published:** 2025-06-09

**Authors:** Jing Guo, Yian Gu

**Affiliations:** 1 School of Public Health, Zhejiang Chinese Medical University, Hangzhou 310053, China; guojingzju@zju.edu.cn; 2 The Taub Institute for Research in Alzheimer’s Disease and the Aging Brain, Columbia University, New York, NY 10032, USA; 3 The Department of Neurology, Columbia University, New York, NY 10032, USA; 4 The Gertrude H. Sergievsky Center, Columbia University, New York, NY 10032, USA; 5 The Department of Epidemiology, Joseph P. Mailman School of Public Health, Columbia University, New York, NY 10032, USA

**Keywords:** nutrition, dementia, cohort study, epidemiology

## Abstract

**Background/Objectives:** Evidence on associations between nutrients and dementia risk is limited and inconsistent. We aimed to systematically examine associations between 101 dietary nutrients and dementia incidence with a nutrient-wide association study (EWAS). **Methods**: We analyzed data from 6280 participants aged 50 years and older from the Health and Retirement Study. Levels of nutrient intake were measured with the food frequency questionnaire. Dementia status was assessed with the Lang–Weir Classification of Cognitive Function. In the EWAS analysis, the Cox proportional hazards regression model was used to estimate associations between each nutrient and dementia incidence, adjusting for multiple comparisons with a false discovery rate (FDR) of 0.05. Nutrients passing the EWAS selection were simultaneously included in the elastic net (ENET) regression model to construct a composite nutrient score (CNS), which was calculated as a weighted sum of the nutrients in the ENET regression model. **Results**: Over a mean (SD) follow-up of 6.76 (2.14) years, 495 individuals with incident dementia were identified. The results suggested that six nutrients were associated with increased dementia risks and five with decreased dementia risks. Compared with participants at the first tertile of CNS, individuals at the second (hazard ratio [HR] = 1.43, 95% confidence interval [CI] = 1.11 to 1.84) and third tertiles (HR = 1.80, 95% CI = 1.42 to 2.27) had increased risks of dementia. Furthermore, CNS-dementia associations were stronger in females than in males. **Conclusions**: We found that 11 dietary nutrients and their combinations were associated with dementia risks in middle-aged and older adults. Interventional studies with nutrients were warranted to confirm our findings.

## 1. Introduction

Dementia affected about 57 million individuals in 2019, and the number will triple by 2050 [1]. Dementia is regarded as a leading cause of disability, dependency, and death in older adults [2]. Given the lack of effective medical treatments, identifying modifiable risk factors to make the preventive strategy has been proposed to reduce the dementia burden [3]. Nutrition is an important environmental factor that has been related to not only the risk of developing dementia but also early pathological changes of dementia before clinical symptoms emerge [4]. It has been reported that oxidative stress and neuroinflammation mediate the pathogenesis and progression of dementia [5,6]. Many dietary nutrients, such as vitamin C, flavonoids, manganese, and *n*-3 fatty acids sourced from fresh fruits, vegetables, nuts, and fish, have antioxidative and anti-inflammatory properties, which may explain their protective roles for cognitive impairment and dementia [7,8]. Associations between certain nutrients (e.g., flavonoids, vitamins, folate, and some lipids) and dementia existed, but the evidence was limited and inconclusive [7]. Inconsistent findings might be caused by the differences in sample sizes, study designs, nutrient assessments, and populations.

Many studies only focused on associations between one or several nutrients and dementia risks and lacked a comprehensive and systematic evaluation [7]. Investigating one or several dietary factors at a time cannot capture the synergistic effects of exposures. The single-exposure approach is prone to generate overestimated effects and increased type I errors due to the interconnected nature of risk factors [9]. In addition, many existing studies selected nutrients, foods, or dietary components for the study based on a priori hypotheses about their beneficial or detrimental roles in cognition. These hypotheses are often generated from evidence about other diseases or general health outcomes, which may not translate directly to dementia or brain health. The nutrient-wide association study (NWAS) approach takes a similar strategy to that of a genome-wide association study in which associations of each risk factor were separately examined and adjusted for multiple comparisons by calculating the false discovery rate (FDR) [10]. The NWAS is a hypothesis-free and data-driven strategy that allows for systematic examination of associations of multiple nutrients and health outcomes, to validate previously established risk factors with reduced bias, and to find novel risk factors [10]. This approach has been applied to estimate dietary risk associations for blood pressure [11] and cancers [12,13], but not for dementia.

Based on the data from the Health and Retirement Study (HRS), we aimed to use the NWAS strategy to comprehensively identify dementia-associated nutrients. We also aimed to estimate the joint effects of identified nutrients by creating composite scores.

## 2. Materials and Methods

### 2.1. Study Design and Participants

The HRS study is an ongoing national longitudinal study that has been conducted biennially since 1992 among US community dwellers aged 50 years and older, with a high response rate of about 85% during follow-up [14]. The HRS study collects a wide range of information covering demographic characteristics, health status, lifestyle, employment, and economic levels [14]. Information on food consumption and nutrition intake was collected in the Health Care and Nutrition Study (HCNS), which surveyed a subsample of HRS participants (*n* = 12,418) in 2013 and obtained a response rate of 65% (*n* = 8073). The HRS study was approved by the University of Michigan Institutional Review Board. The written informed consent was collected from all participants.

Among 8073 participants from HCNS, 37 individuals who completed less than 3% of the food frequency questionnaire (FFQ) and one individual on tube feeding were excluded (Supplementary Figure S1). We also excluded participants who consumed implausible daily energy intakes (<800 or >4200 kcal/day for men, and <600 or >3500 kcal/day for women) (*n* = 625) [15], were younger than 50 years (*n* = 222), had prevalent dementia (*n* = 511), had no assessment of dementia status during the follow-up period (*n* = 239), had unreasonable follow-up time (<0 or >10 years, *n* = 5), or had missing data on covariates (*n* = 153). Thus, a total of 6280 participants were included in the data analysis.

### 2.2. Assessment of Dietary Nutrients

A validated 163-item FFQ was mailed to a random sample of HRS participants in 2013 to assess food and beverage consumption. Participants were asked how often, on average, they consumed the food and beverage at a specified amount during the past twelve months. Each FFQ item included one fixed portion size option. The average daily intake of each food and nutrition item was calculated by the HRS team. The energy-adjusted levels of nutrients were estimated as the standardized residuals produced by regressing the nutrient exposure on total energy [16]. Levels of 203 nutrients were contained in the HCNS data. We excluded one of the paired nutrients with a high correlation (r > 0.9), and 7 nutrients with “near zero variance” of which the frequency ratio of the most common value to the second most common value was more than 95/5, or the percentage of distinct values out of the number of total samples less than 10%, retaining a total of 101 nutrients.

### 2.3. Assessment of Cognition and Dementia

The dementia status, assessed with the Langa–Weir Classifications, was available in a researcher-contributed data set [17]. The cognitive function was quantified with the modified Telephone Interview for Cognitive Status (TICS-m), which tested the abilities of immediate and delayed recall, serial subtraction by 7, and counting backward. Higher scores of TICS-m (0 to 27 points) indicated better cognitive performance. Individuals whose TICS-m scores were less than 6 points were defined as having dementia according to the criteria of Lang–Weir Classification of Cognitive Function [18].

The cognitive abilities of HRS respondents were also assessed by the proxy respondents referring to memory levels (excellent, very good, good, fair, poor), limitations in instrumental activities of daily living (cooking, taking medication, shopping, using phone, and managing money), and status of cognitive impairment (no, maybe, yes). Higher total scores of proxy assessment (0 to 11 points) indicated poorer cognitive function. Participants with dementia were also identified when having proxy assessment scores of 6 or higher [18].

### 2.4. Covariates

Covariates were selected based on associations between dietary habits and dementia in previous studies [19,20,21]. Structured questionnaires were used to collect information on age (in years), sex (male, female), race and ethnicity (White or Caucasian, Black or African American, or other), and educational attainment (in years). Marital status was categorized into married or partnered; separated, divorced, or widowed; and never married. Smoking status contained never, current, and past smoking. Levels of body mass index (BMI, kg/m^2^) were self-reported and divided into underweight (<18.5), normal weight (18.5 to <25), overweight (25 to <30), and obese (≥30). Physical activity scores were calculated by summing the weighted frequency of moderate and vigorous activities, with higher scores meaning higher levels of physical activity [22]. The comorbidity burden (0 to 8 points) was quantified by summing the total number of self-reported hypertension, diabetes, heart diseases, stroke, lung diseases, cancer, psychological problems, and arthritis. Disability in physical function was defined as having any difficulties in daily living activities (bathing, dressing, toileting, eating, transferring, and walking across a room). The dietary calories (in kcal/day) were also used as a covariate.

### 2.5. Statistical Analysis

Baseline characteristics of participants by dementia status were compared with a *t*-test (continuous data with normal distribution), Wilcoxon rank-sum test (continuous data with skewed distribution), or χ^2^ test (categorical data). The normality of continuous variables was checked with the Kolmogorov–Smirnov test. In the single-nutrient approach, associations between each nutrient and dementia risks were separately examined with Cox proportional hazards regression models, adjusting for covariates of age, sex, race or ethnicity, educational attainment, marital status, smoking, BMI categories, comorbidity, physical activity, disability, and calorie intake. The Weibull accelerated failure time regression model was used if the hazard proportionality was violated (*p* values of Schoenfeld residuals test <0.0001 [23]) in the Cox regression model. The *p* value of a single nutrient was adjusted by the FDR method to obtain a q value in consideration of multiple comparisons.

In the multiple-nutrient approach, nutrients with q values less than 0.05 were simultaneously included in the elastic net (ENET) regression model to predict the risks of incident dementia. The ENET model is a regularized regression combining the Ridge and Lasso penalties to improve prediction capability and avoid overfitting. The covariates mentioned above were forced into the ENET model. A 10-fold cross-validation was used to estimate parameters for minimal prediction errors. To summarize the effects of exposure to multiple nutrients, a composite nutrient score (CNS) was calculated as a weighted sum of the nutrients selected by the ENET model, with weights equal to the coefficients of the model. Details in the construction of the ENET model and calculation of CNS are shown in Supplementary Table S1. Associations of continuous CNS and tertiles of CNS with dementia risks were examined with the Cox proportional hazards regression models. The Cox restricted cubic spline model with three knots at the 10th, 50th, and 90th percentiles of CNS was used to analyze dose-response relationships between CNS and dementia.

We performed a few exploratory analyses. First, we examined the association between CNS and cognitive decline with a mixed-effects linear regression model, of which CNS, time, and a product term of CNS × time were used as predictors, and cognitive scores were used as the dependent variable. A significant interaction term of CNS × time indicates differential rates of change in cognition as a function of CNS exposure. Second, we examined whether any other factor (sex, race/ethnicity, smoking, BMI, etc.) can modify the association of CNS with dementia. The product-term interactions between CNS and each covariate were separately analyzed with the Wald test. Association analyses were stratified by the covariates, which showed significant interactions with CNS. Finally, the apolipoprotein E (*APOE*) *ε*4 status (with and without *APOE ε*4 alleles) was additionally adjusted for the estimation of CNS–dementia associations in a subset of participants with available *APOE ε*4 information (*n* = 5285).

All data analyses were performed with R, version 4.2.1. Two-sided *p* < 0.05 was statistically significant.

## 3. Results

### 3.1. Baseline Characteristics of Participants

Compared with participants excluded, the included individuals were more likely to be older, males, White or Caucasia, and less educated (Supplementary Table S2).

The 6280 participants included in the study had a mean (SD) follow-up of 6.76 (2.14) years, with 42,440 person-years in total. A total of 495 participants developed incident dementia. As shown in Table 1, the mean (SD) age at baseline was 66.88 (10.13) years, and about 40% of the participants were males. The majority (accounting for 78.36%) of participants were White or Caucasian. Compared with individuals without an incident of dementia, the newly identified cases were more likely to be older, Black or African American, less educated, separated or divorced, or widowed, and physically dependent. Furthermore, individuals with incident dementia had a heavier burden of comorbidity, lower levels of physical activity, higher levels of dietary calorie intake, and lower proportions of obesity.

Participants with a higher CNS were more likely to be older, Black or African American, less educated, separated or divorced, or widowed, current smokers, and disabled (Supplementary Table S3). Individuals with a higher CNS also had a heavier burden of comorbidity, lower levels of physical activity and cognitive score, and a higher proportion of developed incident dementia.

### 3.2. Associations Between Single Nutrient and Dementia

**Figure 1:**
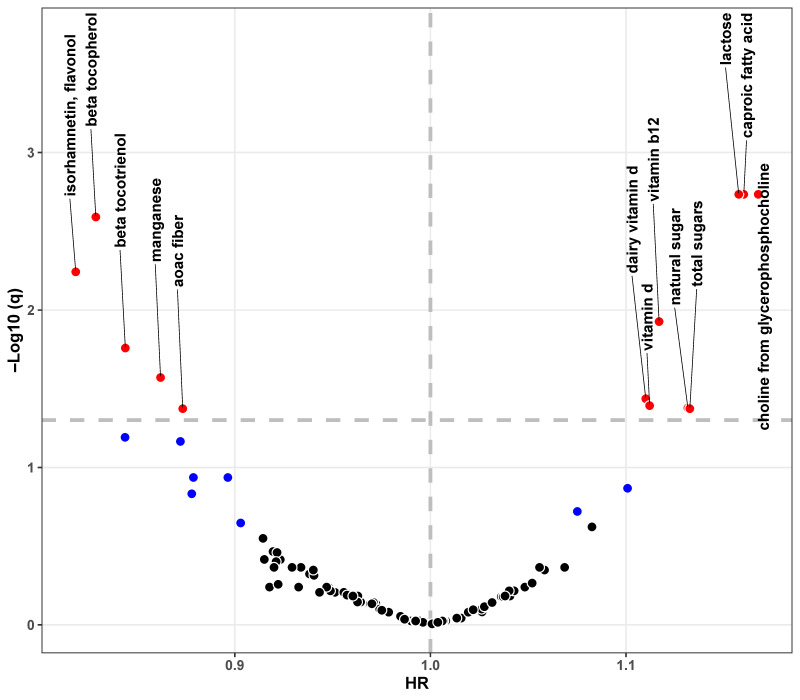
Volcano plot showing results of NWAS analysis regarding associations between 101 nutrients and dementia risks. The x-axis represents hazard ratio for each nutrient per 1-SD increase in consumption. The y-axis represents q values (FDR-adjusted *p* values) in -log10 scale. Associations between each nutrient and dementia risks were separately estimated with the fully adjusted Cox proportional hazards regression model. The horizontal and vertical dashed lines represent the threshold of q values at 0.05 and hazard ratios at 1, respectively. Scatters in black, blue, and red mean the *p* values ≥ 0.05, *p* values < 0.05 and q values ≥ 0.05, and q values < 0.05, respectively. Abbreviation: CI, confidence interval; FDR, false discovery rate; HR, hazard ratio; SD, standard deviation.

In the single-nutrient approach, 21 out of 101 nutrients were found to be significantly associated with dementia risks (Figure 1, Supplementary Table S4). Among them, 11 nutrients suggested potentially protective effects (hazard ratio [HR] < 1, *p* < 0.05) and 10 were detrimental (HR > 1, *p* < 0.05). After the FDR adjustment, we found that five nutrients (isorhamnetin, beta tocopherol, beta tocotrienol, manganese, and AOAC fiber) were significantly associated with decreased dementia risks (FDR-adjusted *p* < 0.05) and eight nutrients (choline from glycerophosphocholine, aproic fatty acid, lactose, total sugars, natural sugar, vitamin B12, vitamin D, and dairy vitamin D) were linked to increased dementia risks. Some of these selected nutrients were significantly correlated (Spearman correlation coefficients, −0.29 to 0.81) (Supplementary Figure S2).

### 3.3. Associations Between CNS and Dementia

**Figure 2:**
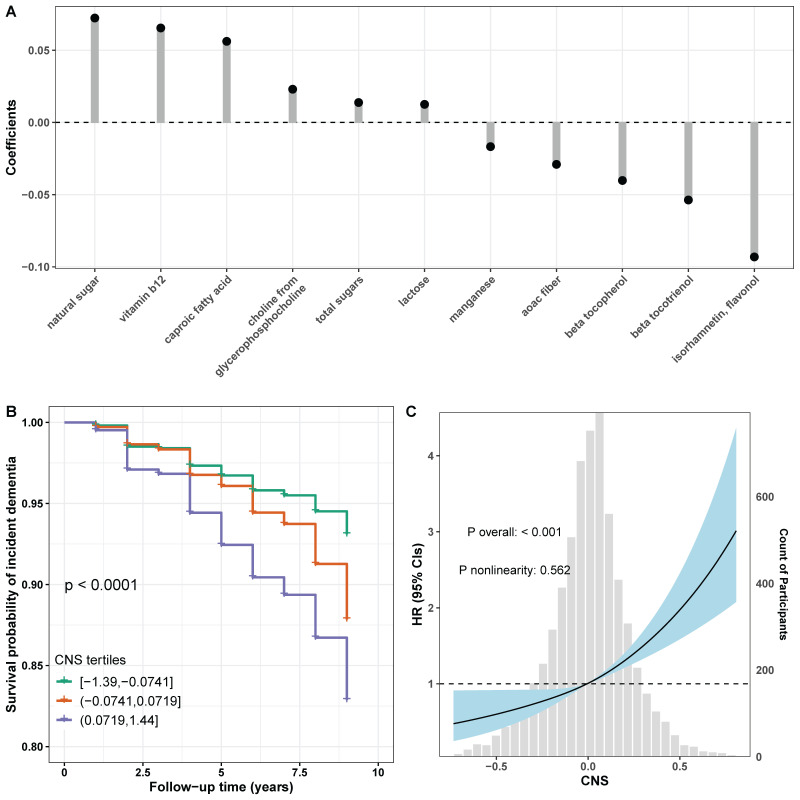
Associations between CNS and dementia risks. (**A**) Coefficients of selected nutrients in the ENET regression model. Thirteen nutrients with q values less than 0.05 from the single-nutrient analysis were simultaneously included in the ENET regression model where all covariates were forced in. Coefficients of dairy vitamin D and total vitamin D were shrunk to zero and not shown. (**B**) Kaplan–Meier curves show the survival probabilities of dementia by CNS tertiles (log-rank test, *p* < 0.001). (**C**) Dose-response relationships between CNS and dementia risks. Restricted cubic splines with three knots at the 10th, 50th, and 90th percentiles of CNS were included in the fully adjusted Cox regression model to estimate the CNS-dementia association. The histogram at bottom shows the distribution of CNS. The solid line and blue area represent the HR and corresponding 95% CI, respectively. Abbreviation: CI, confidence interval; CNS, composite nutrient score; HR, hazard ratio.

In the multiple-nutrient approach, 11 of 13 nutrients were retained in the ENET model, where the coefficients ranged from −0.09 for isorhamnetin to 0.07 for natural sugar (panel A in Figure 2, Supplementary Table S5). Two nutrients of total vitamin D and dairy vitamin D were removed by the ENET model. As shown in Table 2 and panel B in Figure 2, compared with participants with the first tertile (T1) of CNS, those with the second (T2, HR = 1.43, 95% confidence interval [CI] = 1.11 to 1.84, *p* = 0.005) and third (T3, HR = 1.80, 95% CI = 1.42 to 2.27, *p* < 0.001) titles had significantly increased dementia risks (*p* trend < 0.001). The results of the Cox restricted cubic spline model indicated that CNS was positively associated with incident dementia, potentially in a linear manner (*p* for overall association < 0.001, *p* for nonlinearity = 0.562) (panel C in Figure 2).

### 3.4. Exploratory Analysis

As shown in Supplementary Table S6, participants with higher levels of CNS had significantly lower cognitive scores at baseline (CNS T3 vs. T1, β = −0.532, 95% CI = −0.983 to −0.080, *p* = 0.021) and faster cognitive decline over follow-up (CNS T3 vs. T1, β = −0.072, 95% CI = −0.137 to −0.007, *p* = 0.031).

We found that interactions between sex and CNS were significant (*p* interaction = 0.017), and the CNS-dementia associations seemed to be stronger in females than in males (Table 2). No other factors moderated the relationship between CNS and dementia risk.

Similar associations between CNS and dementia risks were found when additionally adjusting for the status of *APOE ε*4 alleles (Supplementary Table S7).

## 4. Discussion

In this prospective cohort study of 6280 US adults older than 50 years, we used the NWAS approach to systematically evaluate associations of dietary intakes of 101 nutrients with dementia risk. The results suggested that dementia risks were positively associated with eight nutrients and inversely associated with another five nutrients after the FDR adjustment. We constructed a new dietary pattern, the CNS, based on the key nutrients identified from NWAS, and found it was significantly associated with increased risks of dementia and faster cognitive decline. Our findings shed light on the prevention and control of dementia from a nutritional perspective.

Our findings confirmed the previously reported associations between sugar intake and increased risks of dementia. Data from the UK Biobank indicated that dietary total sugar was positively associated with Alzheimer’s disease (AD) [24]. Positive associations between sugar intake and dementia were also found among US older adults [25].

Milk is rich in caproic fatty acid, glycerophosphocholine, lactose, and vitamin D, and our findings showed positive associations between these clustered nutrients and dementia risks. A prospective study of 13,751 participants of the Atherosclerosis Risk in Communities cohort found that individuals who consumed higher levels of milk at midlife had faster cognitive decline over a 20-year follow-up [26]. Data from older Japanese adults showed positive associations between total dairy intake or milk intake frequency with dementia risks; however, inverse associations between yogurt consumption and dementia risks were also found [27]. Other studies have shown that dietary vitamin D intake was associated with a reduced risk of AD [28]. Inconsistent results might be caused by different dietary habits, study designs, and populations in previous studies. In the current study, intakes of dairy vitamin D and total vitamin D were identified by the single-nutrient analysis but were removed by the ENET model. It is possible that other nutrients existing in vitamin D-rich foods might have a stronger association with dementia. As mentioned above, dairy vitamin D and total vitamin D had milk and dairy as similar food sources and were highly correlated with lactose, caproic fatty acids, and glycerophosphocholine (Supplementary Figure S2), which were all retained in the ENET model.

Interestingly, all three nutrients (lactose, caproic fatty acids, and glycerophosphocholine) were associated with an increased risk of dementia. Few studies have examined the independent role of lactose intake on dementia risk or cognitive health in older adults. A cross-sectional study on 1209 participants aged  ≥  60 and found that higher intake of lactose was associated with lower cognitive scores [29]. A recent large prospective study of 4586 participants of the Women’s Health Initiative—Dietary Modification Trial found that dietary lactose intake was associated with an increased risk of dementia [30]. While the exact mechanisms are unclear, studies have shown that lactose can be metabolized into d-galactose, which may contribute to increased oxidative stress and inflammation [31].

The intake of caproic fatty acid, a saturated short-chain fatty acid (SCFA), is mainly from milk fat and cocoa oil. The SCFAs can be produced by gut microbiota via the fermentation of dietary fiber ingested and are regarded as important components of the gut-brain axis against AD [32]. In line with our findings, a cohort study of 2612 older adults reported that dietary intake of SCFAs was associated with a higher dementia incidence (tertile 3 vs. tertile 1, relative risk = 1.52, 95% CI = 1.19 to 1.95), although this association was attenuated to be nonsignificant when adjusting for multiple covariates [33]. The SCFAs may have the capabilities to modulate neuroinflammation, maintain the blood-brain barrier, interfere with amyloid protein formation, and regulate brain metabolism [34]. However, other recent evidence suggested that SCFAs may exert detrimental effects on the pathogenesis of AD, including neuroinflammation, insulin resistance, and brain amyloid deposition [34]. As a result, the health effects of SCFAs on the development of dementia are complicated and inconsistent, for solid conclusions.

With regard to the association between glycerophosphocholine and dementia, current evidence has been mixed. Results from the UK Biobank showed a U-shaped relationship between glycerophosphocholine intake and dementia incidence, with a significantly inverse association at the moderate intake of glycerophosphocholine [35]. Original levels of glycerophosphocholine intake were used in the UK Biobank study [35], which was different from the standardized residuals produced by regressing the nutrient on total energy in our study. In the present study, glycerophosphocholine-dementia associations tended to be linear (Supplementary Figure S3).

We found vitamin-B12-associated increased risks of dementia. It has been reported that intakes of vitamin B12 were not significantly associated with risks of mild cognitive impairment and dementia in US postmenopausal women [36] and French older adults [37]. Data from the UK general population showed significantly inverse associations of dietary vitamin B12 (quartile 4 vs. quartile 1, HR = 0.77, 95% CI = 0.64 to 0.93) with risks of AD [38]. Vitamin B12 is one of the homocysteine-related vitamins that may have a protective role on AD via its ability to reduce circulating homocysteine levels. However, a recent study of healthy older adults found that both low and high levels of vitamin B12 in blood were associated with poorer cognitive function and increased levels of Tau protein [39], which forms pathologic fibrillar aggregates that are linked to neuronal cell death and neurodegeneration [40]. Another study found that a dietary pattern low in B12 was associated with reduced AD risk, suggesting a potential positive link between higher B12 and higher AD risk [41]. However, since vitamin B12 is primarily found in meat and dairy, which are also high in saturated fatty acids and other nutrients linked to AD risk [42], its independent role remains unclear. The exact role of vitamin B12 in dementia should be further investigated.

A higher dietary intake of isorhamnetin, a type of flavonol, was linked to decreased dementia risks in the present study. Similarly, associations between isorhamnetin intake and AD risks were significantly inverse (the fifth vs. first quintiles: HR = 0.62, 95% CI = 0.39 to 0.98) in another cohort study of 921 older adults [43]. Isorhamnetin has the capability to attenuate the Aβ-triggered secretion of interleukin-6, an inflammatory biomarker, and exerts its neuroprotective effects by mediating neuroinflammation [44]. Furthermore, we found decreased dementia risks associated with higher intakes of β-tocopherol and β-tocotrienol which are natural compounds of vitamin E. Data from 1041 participants aged 65 years and older suggested that an increase of 5 mg/day in vitamin E intake was associated with a decreased risk of AD of 26% (HR = 0.74, 95% CI = 0.62 to 0.88), however associations of β-tocopherol intake were not statistically significant [45]. Another cohort study reported that elderly adults with higher serum levels of β-tocotrienol had lower risks of developing cognitive impairment [46]. Evidence from experiments in vitro and in animal models suggested antioxidant and anti-inflammatory properties of tocopherols and tocotrienols; however, their application in dementia treatment is still under debate due to the inconsistent results from observational studies [47].

Manganese-associated decreased risks of dementia were found in this study. In line with our findings, two cross-sectional studies reported decreased odds of cognitive impairment and better cognitive performance with higher dietary intake of manganese in older adults [48,49]. As a result, manganese–dementia associations need to be confirmed in other prospective cohort studies. Furthermore, we found inverse associations between dietary fiber and dementia risk. It has been reported that dietary fiber potentially modulates brain function via the “microbiota–gut–brain” axis [50]. Epidemiological evidence also showed that higher dietary fiber intake was linked with slower cognitive decline [51] and decreased risks of dementia [52].

The collinearity problem might be produced if all the 13 nutrients from the single-nutrient analysis were included simultaneously in the same Cox regression model, which could be overcome by the ENET model. We applied the ENET model, which reduced the redundancy by removing highly correlated nutrients, ranked the importance of each selected nutrient, and estimated the combined effects of a panel of 11 nutrients retained in the ENET model. Higher levels of CNS derived from ENET were linked to increased risks of dementia. Thus, the CNS represents a nutrient pattern that is parsimonious, informative, and relevant for the outcome, highlighting the innovation and importance of assessing the health effects of nutrient mixtures in epidemiological studies.

While CNS was significantly associated with dementia risk in both men and women, our results showed stronger CNS-dementia associations in women than in men. Similarly, data from a previous study indicated that poorer diet quality was more strongly associated with all-cause mortality in women compared with men [53]. The results of a meta-analysis study indicated that women with diabetes had a 19% higher risk for vascular dementia than men [54]. Mid-life hypertension was associated with increased risks of dementia in late life in women but not men [55]. Evidence from animal experiments suggested that AD mouse models fed with a high-fat diet displayed poorer spatial memory, more deficits in activities of daily living, more amyloid beta accumulation, and severe glucose intolerance in females than in males [56]. It has been proposed that men and women have differences in nutrient intake and metabolism, diet and gut-microbiome interactions, genetics, and hormones, leading to different health outcomes throughout life [57]. Our results showed that levels of CNS were not significantly different between men and women (*p* = 0.633), suggesting that sex differences in CNS–dementia associations might be due to the differences in biological mechanisms rather than nutrient intakes.

Some limitations should be noted. First, the dementia status was not assessed according to standard clinical guidelines. Furthermore, the dementia status of participants might be misclassified if the proxy respondents had poor cognition. Second, the self-reported FFQ and BMI might induce recall bias, especially among the participants with poor cognitive function. Third, participants were middle-aged and older adults living in the US, which might have precluded the generalization of our findings to other populations with different dietary habits. Fourth, other dementia-related factors (e.g., genetics and air pollution) have not been adjusted in regression models. Fifth, the potential biological mechanisms of our findings have not been fully elucidated. Furthermore, linear associations between nutrients and dementia risks were estimated in our data analysis, which might ignore the nutrients that had nonlinear relationships with dementia.

However, our study has many advantages. The nationally representative survey with a large sample size allows us to conduct data analysis, especially stratified analysis, with sufficient statistical power. More importantly, we used data-driven methods to identify key nutrients for cognitive health and further evaluate their relative importance. Our results are independent of a priori hypotheses, allowing us to identify novel nutrients, such as lactose and glycerophosphocholine, that could have been overlooked in traditional methods that typically focus on common nutrients only, such as vitamin D.

## 5. Conclusions

Our data provided comprehensive evidence for associations between nutrient profiles and dementia incidence in middle-aged and older adults. The present study identified six protective and five detrimental nutrients for dementia and confirmed a significantly positive association between the combinations of these nutrients and dementia risks. Our findings provided novel clues to enrich the nutrient-related strategies for dementia prevention. Interventional studies are warranted to validate our results, and the corresponding mechanisms need to be explored in the future.

## Figures and Tables

**Table 1:** Baseline characteristics by dementia status among all participants.

Characteristics	Total (*n* = 6280)	Non-Demented (*n* = 5785)	Incident (*n* = 495)	*p* Value
Age (years), mean (SD)	66.88 (10.13)	66.28 (9.87)	73.80 (10.49)	<0.001
Sex, *n* (%)				0.810
Male	2544 (40.51)	2346 (40.55)	198 (40.00)	
Female	3736 (59.49)	3439 (59.45)	297 (60.00)	
Race/ethnicity, *n* (%)				<0.001
White/Caucasian	4921 (78.36)	4564 (78.89)	357 (72.12)	
Black/African American	929 (14.79)	822 (14.21)	107 (21.62)	
Other	430 (6.85)	399 (6.90)	31 (6.26)	
Years of education, mean (SD)	13.21 (2.79)	13.32 (2.71)	11.93 (3.34)	<0.001
Marital status, *n* (%)				<0.001
Married/partnered	4232 (67.39)	3955 (68.37)	277 (55.96)	
Separated/divorced/widowed	1791 (28.52)	1594 (27.55)	197 (39.80)	
Never married	257 (4.09)	236 (4.08)	21 (4.24)	
Smoking, *n* (%)				0.682
Never	2875 (45.78)	2654 (45.88)	221 (44.65)	
Past	2678 (42.64)	2458 (42.49)	220 (44.44)	
Current	727 (11.58)	673 (11.63)	54 (10.91)	
BMI categories, *n* (%)				<0.001
Underweight	67 (1.07)	54 (0.93)	13 (2.63)	
Normal weight	1658 (26.40)	1499 (25.91)	159 (32.12)	
Overweight	2330 (37.10)	2130 (36.82)	200 (40.40)	
Obese	2225 (35.43)	2102 (36.34)	123 (24.85)	
Number of comorbidities, mean (SD)	2.01 (1.43)	1.97 (1.42)	2.44 (1.47)	<0.001
Score of physical activity, mean (SD)	8.19 (6.66)	8.39 (6.68)	5.91 (6.00)	<0.001
Disability, *n* (%)				<0.001
No	5462 (86.97)	5096 (88.09)	366 (73.94)	
Yes	818 (13.03)	689 (11.91)	129 (26.06)	
Dietary calories (kcal/day), mean (SD)	1770.67 (660.22)	1764.00 (655.44)	1848.58 (709.85)	0.011

Abbreviation: BMI, body mass index; SD, standard deviation.

**Table 2:** Associations between CNS and risks of dementia.

		Model 1 ^a^		Model 2 ^b^	
Subpopulation	No. of Events/Person-Years	HR (95% CI)	*p* Value	HR (95% CI)	*p* Value
All (*n* = 6280)					
CNS categories ^c^					
T1 (*n* = 2094)	103/14,583	Reference		Reference	
T2 (*n* = 2093)	157/14,248	1.45 (1.13, 1.86)	0.004	1.43 (1.11, 1.84)	0.005
T3 (*n* = 2093)	235/13,609	1.89 (1.50, 2.39)	<0.001	1.80 (1.42, 2.27)	<0.001
Continuous CNS	495/42,440	4.18 (2.94, 5.92)	<0.001	3.55 (2.49, 5.05)	<0.001
Males (*n* = 2544)					
CNS categories ^c^					
T1 (*n* = 830)	43/5651	Reference		Reference	
T2 (*n* = 842)	66/5726	1.38 (0.94, 2.05)	0.104	1.32 (0.89, 1.96)	0.162
T3 (*n* = 872)	89/5627	1.65 (1.14, 2.39)	0.007	1.53 (1.05, 2.21)	0.026
Continuous CNS	198/17,004	2.65 (1.49, 4.71)	0.001	2.23 (1.24, 4.01)	0.007
Females (*n* = 3736)					
CNS categories ^c^					
T1 (*n* = 1264)	60/8932	Reference		Reference	
T2 (*n* = 1251)	91/8522	1.49 (1.07, 2.07)	0.018	1.55 (1.11, 2.16)	0.010
T3 (*n* = 1221)	146/7982	2.06 (1.52, 2.78)	<0.001	2.01 (1.48, 2.72)	<0.001
Continuous CNS	297/25,436	5.47 (3.52, 8.49)	<0.001	4.58 (2.94, 7.13)	<0.001

Abbreviations: CNS, composite nutrient score; HR, hazard ratio; T1–T3, the 1st to the 3rd tertiles. ^a^ Model 1 was adjusted for age, sex, race/ethnicity, years of education, and dietary calories. ^b^ Model 2 was adjusted for covariate sin Model 1 plus marital status, smoking, BMI categories, physical activity, comorbidity, and disability. ^c^ CNS was categorized into three groups of T1 (≥−1.390 to ≤−0.074), T2 (>−0.074 to ≤0.072), and T3 (>0.072 to ≤1.44) according to tertiles.

## Data Availability

All data used in the present study are publicly available on the website of the Health and Retirement Study (https://hrs.isr.umich.edu/about (accessed on 1 January 2024)).

## Supplementary Materials

The following supporting information can be downloaded at: https://www.mdpi.com/article/10.3390/nu17121960/s1, Figure S1: Flow chart of participant selection; Table S1: Construction of ENET model and calculation of CNS; Table S2: Comparison of baseline characteristics between included and excluded participants; Table S3: Baseline characteristics by CNS tertiles; Table S4: Associations between each nutrient and dementia risks; Figure S2: Correlations between selected nutrients; Table S5: Coefficients of selected nutrients based on the elastic net penalized Cox regression model; Table S6: Associations between CNS and cognitive decline with mixed-effects linear regression model; Table S7: Associations between CNS and risks of dementia when additionally adjusting for the status of *APOE ε*4 alleles; Figure S3: Dose-response associations between selected nutrients and dementia risks. References [58,59,60] are cited in the supplementary materials.

## Abbreviations

The following abbreviations are used in this manuscript:

AD	Alzheimer’s disease
BMI	Body mass index
CI	Confidence interval
CNS	Composite nutrient score
ENET	Elastic net
EWAS	Nutrient-wide association study
FDR	False discovery rate
FFQ	Food frequency questionnaire
HCNS	Health Care and Nutrition Study
HR	Hazard ratio
HRS	Health and Retirement Study
SCFA	Saturated short-chain fatty acid
TICS-m	Modified Telephone Interview for Cognitive Status
