# Supplementary material for: Nutrient-Wide Association Study for Dementia Risks: A Prospective Cohort Study in Middle-Aged and Older Adults
Source: Nutrients. 2025 Jun 9;17(12):1960. doi: 10.3390/nu17121960 (PMC12196508; doi:10.3390/nu17121960)
Supplement: Supplementary file 1 [file nutrients-17-01960-s001.zip › nutrients-3648547-supplementary.pdf]

Supplementary materials

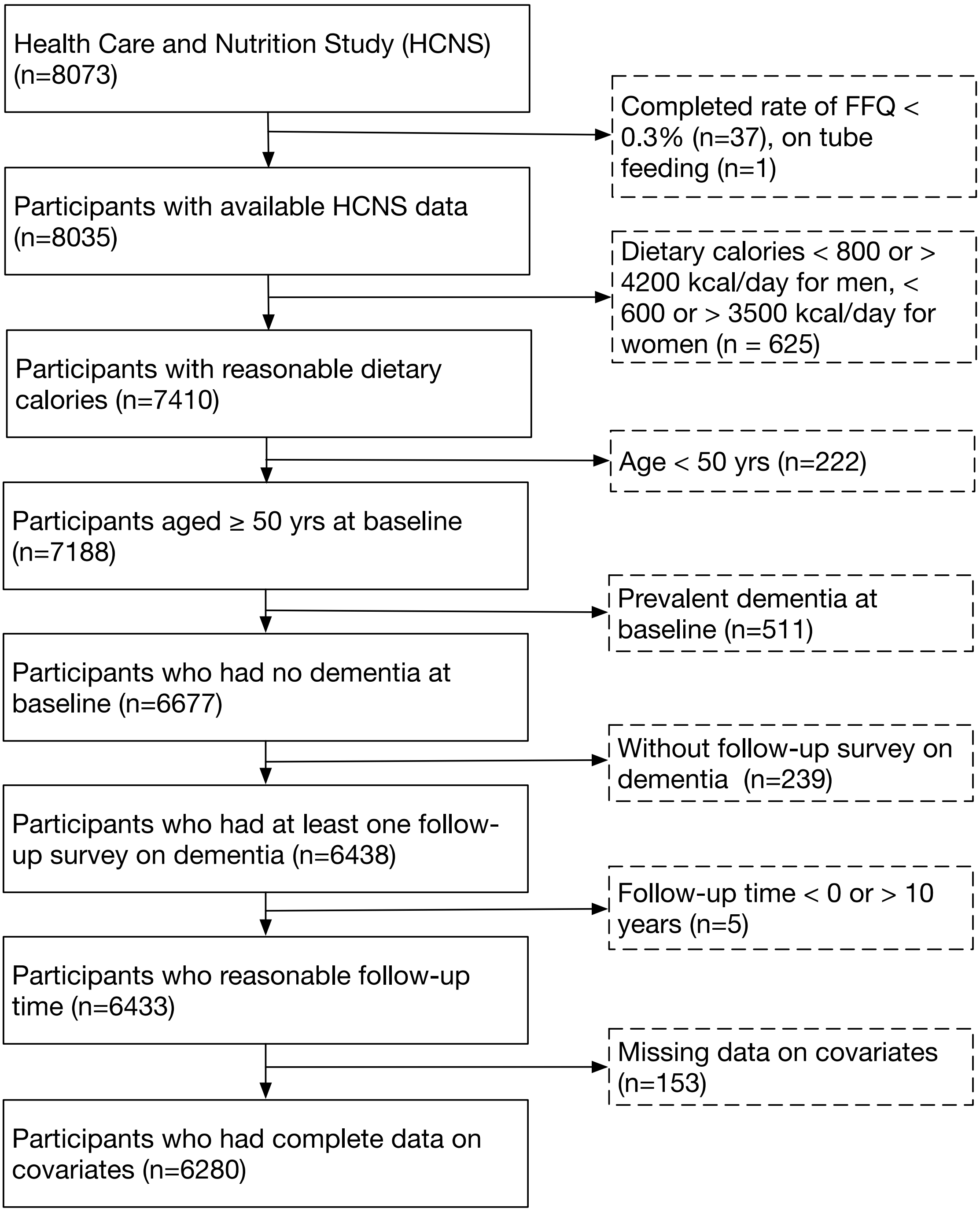

Figure S1. Flow chart of participant selection.

Table S1. Construction of ENET model and calculation of CNS.

Construction of ENET model	The ENET model is a hybrid of ridge regression and LASSO regularization. The ENET model is penalized with both the L1-norm and L2-norm to effectively shrink coefficients (like in ridge regression) and to set some coefficients to zero (as in LASSO). Details in the algorithm of ENET for regularized Cox models could be found elsewhere [1-3].
Calculation of CNS	For the i-th participant: $$CNS_i = \sum_{j=1}^{p} \hat{\beta}_j E_i^j$$ where E_i^j ($j = 1, \ldots, p$) is the z-score of the j-th nutrient and $\hat{\beta}_j$ is the beta coefficient (weight) of the j-th nutrient from the ENET model.

Abbreviations: ENET, elastic net; CNS, composite nutrient score.

References

1. Tay, J.K.; Narasimhan, B.; Hastie, T. Elastic Net Regularization Paths for All Generalized Linear Models. Journal of Statistical Software 2023, 106, 1 - 31, doi:10.18637/jss.v106.i01.
2. Simon, N.; Friedman, J.H.; Hastie, T.; Tibshirani, R. Regularization Paths for Cox's Proportional Hazards Model via Coordinate Descent. Journal of Statistical Software 2011, 39, 1 - 13, doi:10.18637/jss.v039.i05.
3. Zou, H.; Hastie, T. Regularization and Variable Selection Via the Elastic Net. Journal of the Royal Statistical Society Series B: Statistical Methodology 2005, 67, 301-320, doi:10.1111/j.1467-9868.2005.00503.x.

Table S2. Comparison of baseline characteristics between included and excluded participants.

Characteristics	Total (n =7187)	Excluded (n = 907)	Included (n = 6280)	*P* value
Age (years), mean (SD)	67.44 (10.39)	71.35 (11.34)	66.88 (10.13)	< 0.001
Sex, n (%)				< 0.001
Male	2971 (41.40)	427 (47.66)	2544 (40.51)	
Female	4205 (58.60)	469 (52.34)	3736 (59.49)	
Race/ethnicity, n (%)				< 0.001
White/Caucasian	5545 (77.45)	624 (70.99)	4921 (78.36)	
Black/African American	1112 (15.53)	183 (20.82)	929 (14.79)	
Other	502 (7.01)	72 (8.19)	430 (6.85)	
Years of education, mean (SD)	12.98 (2.98)	11.32 (3.72)	13.21 (2.79)	< 0.001
Marital status, n (%)				< 0.001
Married/partnered	4726 (66.45)	494 (59.38)	4232 (67.39)	
Separated/divorced/widowed	2095 (29.46)	304 (36.54)	1791 (28.52)	
Never married	291 (4.09)	34 (4.09)	257 (4.09)	
Smoking, n (%)				0.050
Never	3206 (45.32)	331 (41.69)	2875 (45.78)	
Past	3032 (42.86)	354 (44.58)	2678 (42.64)	
Current	836 (11.82)	109 (13.73)	727 (11.58)	
BMI categories, n (%)				0.036
Under weight	79 (1.11)	12 (1.48)	67 (1.07)	
Normal weight	1906 (26.87)	248 (30.50)	1658 (26.40)	
Overweight	2625 (37.01)	295 (36.29)	2330 (37.10)	
Obese	2483 (35.01)	258 (31.73)	2225 (35.43)	
Number of comorbidities, mean (SD)	7.91 (6.67)	5.78 (6.27)	8.19 (6.66)	< 0.001
Score of physical activity, mean (SD)	2.08 (1.46)	2.56 (1.58)	2.01 (1.43)	< 0.001
Disability, n (%)				< 0.001
No	6044 (85.00)	582 (70.04)	5462 (86.97)	
Yes	1067 (15.00)	249 (29.96)	818 (13.03)	
Dietary calories (kcal/day), mean (SD)	1781.65 (672.72)	1857.66 (749.56)	1770.67 (660.22)	< 0.001

Abbreviation: BMI, body mass index; SD, standard deviation.

Table S3. Baseline characteristics by CNS tertiles.

Characteristics	Total (n = 6280)	CNS tertiles [a]			
		T1 (n = 2094)	T2 (n = 2093)	T3 (n = 2093)	*P* value [b]
Age (years), mean (SD)	66.88 (10.13)	65.41 (9.45)	67.22 (10.11)	68.00 (10.62)	< 0.001
Sex, n (%)					0.390
Male	2544 (40.51)	830 (39.64)	842 (40.23)	872 (41.66)	
Female	3736 (59.49)	1264 (60.36)	1251 (59.77)	1221 (58.34)	
Race/ethnicity, n (%)					0.009
White/Caucasian	4921 (78.36)	1642 (78.41)	1668 (79.69)	1611 (76.97)	
Black/African American	929 (14.79)	285 (13.61)	299 (14.29)	345 (16.48)	
Other	430 (6.85)	167 (7.98)	126 (6.02)	137 (6.55)	
Years of education, mean (SD)	13.21 (2.79)	13.47 (2.87)	13.29 (2.64)	12.86 (2.83)	< 0.001
Marital status, n (%)					< 0.001
Married/partnered	4232 (67.39)	1497 (71.49)	1420 (67.85)	1315 (62.83)	
Separated/divorced/widowed	1791 (28.52)	511 (24.40)	594 (28.38)	686 (32.78)	
Never married	257 (4.09)	86 (4.11)	79 (3.77)	92 (4.40)	
Smoking, n (%)					< 0.001
Never	2875 (45.78)	993 (47.42)	942 (45.01)	940 (44.91)	
Past	2678 (42.64)	920 (43.94)	898 (42.90)	860 (41.09)	
Current	727 (11.58)	181 (8.64)	253 (12.09)	293 (14.00)	
BMI categories, n (%)					0.427
Under weight	67 (1.07)	21 (1.00)	21 (1.00)	25 (1.19)	
Normal weight	1658 (26.40)	583 (27.84)	517 (24.70)	558 (26.66)	
Overweight	2330 (37.10)	764 (36.49)	794 (37.94)	772 (36.88)	
Obese	2225 (35.43)	726 (34.67)	761 (36.36)	738 (35.26)	
Number of comorbidities, mean (SD)	2.01 (1.43)	1.88 (1.39)	2.04 (1.41)	2.11 (1.47)	< 0.001
Score of physical activity, mean (SD)	8.19 (6.66)	9.33 (6.67)	7.76 (6.59)	7.49 (6.59)	< 0.001
Disability, n (%)					< 0.001
No	5462 (86.97)	1878 (89.68)	1830 (87.43)	1754 (83.80)	
Yes	818 (13.03)	216 (10.32)	263 (12.57)	339 (16.20)	
Dietary calories (kcal/day), mean (SD)	1770.67 (660.22)	1885.00 (636.45)	1579.42 (618.58)	1847.53 (681.78)	< 0.001
Cognitive score, mean (SD)	15.87 (3.77)	16.42 (3.68)	15.89 (3.78)	15.29 (3.76)	< 0.001
Dementia, n (%)					< 0.001
Non-demented	5785 (92.12)	1991 (95.08)	1936 (92.50)	1858 (88.77)	

Incident	495 (7.88)	103 (4.92)	157 (7.50)	235 (11.23)

Abbreviation: BMI, body mass index; CNS, composite nutrient score; SD, standard deviation; T1 to T3, the first to the third tertiles.

[a] CNS was categorized into three groups of T1 (≥ -1.390 to ≤ -0.074), T2 (> -0.074 to ≤ 0.072), and T3 (> 0.072 to ≤ 1.44) according to tertiles.

[b] Baseline characteristics of participants by CNS tertiles were compared with the χ^2 test for categorical variables and analysis of variance for continuous variables.

Table S4. Associations between each nutrient and dementia risks.

Nutrients [a]	HR (95% CI) [b]	*P* of HR	FDR-adjusted *P* of HR	*P* of Schoenfeld residuals test	Models used
alpha carotene	0.99 (0.91, 1.08)	0.8139787	0.94496377	0.00090784	Cox
acrylamide	0.92 (0.84, 1.02)	0.10296909	0.38518067	0.00015259	Cox
discretionary liquid fats	0.92 (0.84, 1.01)	0.08128641	0.34208033	0.00093095	Cox
added sugar	1.04 (0.95, 1.14)	0.37387474	0.65612053	0.00069442	Cox
alcohol	1.02 (0.93, 1.11)	0.74041034	0.90607823	0.00082778	Cox
aoac fiber	0.87 (0.79, 0.96)	0.00523656	0.04230732	0.00070359	Cox
aspartame	1.03 (0.92, 1.14)	0.639363	0.82789312	0.0008604	Cox
thiamine	0.97 (0.88, 1.07)	0.52688261	0.73909922	0.00079912	Cox
vitamin b12	1.12 (1.05, 1.19)	0.00070298	0.01183353	0.00107713	Cox
pyridoxine	1.04 (0.97, 1.13)	0.2848859	0.60839568	0.00090408	Cox
beta carotene	0.96 (0.87, 1.05)	0.32638173	0.62197273	0.00083561	Cox
beta cryptoxanthin	0.97 (0.89, 1.06)	0.51647455	0.73470323	0.00011874	Cox
betaine, choline derivative	0.97 (0.89, 1.05)	0.48571197	0.72142514	0.00069559	Cox
added bran from wheat, rice, oat, corn	1.01 (0.92, 1.10)	0.8791816	0.94519611	0.00065649	Cox
natural bran	0.94 (0.86, 1.03)	0.16925317	0.47484918	0.00076632	Cox
caffeine	0.90 (0.82, 1.00)	0.04683308	0.22524483	0.00080334	Cox
calcium	1.08 (1.00, 1.15)	0.03769259	0.1903476	0.00046989	Cox
carbohydrates	1.03 (0.94, 1.13)	0.58465291	0.79409046	0.00057423	Cox
cholesterol	1.00 (0.92, 1.09)	0.98312921	0.98637331	0.00042233	Cox
copper	1.01 (0.93, 1.10)	0.76159495	0.90607823	0.00087801	Cox
dairy vitamin d	1.11 (1.04, 1.19)	0.00325963	0.03658025	0.00045961	Cox
dihydrophylloquinone vitamin k1	0.90 (0.82, 0.98)	0.01949382	0.1158162	0.00054353	Cox
vitamin e mg atoco conversion	0.99 (0.90, 1.09)	0.86800073	0.94519611	0.00039656	Cox
epiprogoitrin, glucosinolate	1.00 (0.91, 1.09)	0.94498086	0.96407138	0.00093197	Cox
vitamin e mg atoco food fortification	1.05 (0.97, 1.14)	0.25492565	0.57576689	0.00084914	Cox
lauric fatty acid	1.05 (0.97, 1.14)	0.24567696	0.57576689	0.00038391	Cox
eicosenoic fatty acid	0.94 (0.86, 1.03)	0.17755797	0.48468528	0.00058943	Cox
arachadonic fatty acid	0.96 (0.88, 1.04)	0.31494794	0.62148446	0.00061329	Cox
eicosapentaenoic epa fatty acid	1.02 (0.94, 1.11)	0.65389616	0.83121392	0.00074312	Cox
docosapentaenoic 22:5 fatty acid	0.99 (0.91, 1.08)	0.90594785	0.95313263	0.0008771	Cox
caproic fatty acid	1.16 (1.08, 1.25)	5.4741E-05	0.00184295	0.00040667	Cox
food folate	0.95 (0.87, 1.04)	0.25025132	0.57576689	0.00043338	Cox
total folate mcg, foods+vits	1.01 (0.92, 1.10)	0.85947516	0.94519611	0.0004939	Cox
folic acid	1.05 (0.97, 1.14)	0.20425504	0.54288839	0.00089706	Cox
free choline, choline-contributing metabolite	0.97 (0.88, 1.06)	0.50297022	0.73470323	0.00084951	Cox
fructose	1.03 (0.94, 1.12)	0.55445715	0.76712565	0.00018963	Cox
added germ from wheat	0.99 (0.92, 1.08)	0.88904585	0.94519611	0.00092351	Cox
natural germ	0.91 (0.83, 1.01)	0.06426613	0.28221215	0.00046049	Cox
glucoiberin, glucosinolate	1.00 (0.91, 1.09)	0.93366205	0.96399973	0.00092269	Cox

glucobrassicanapin, glucosinolate	0.98 (0.91, 1.07)	0.70803467	0.88285805	0.00092725	Cox
glucocheirolin, glucosinolate	1.04 (0.97, 1.12)	0.28913854	0.60839568	0.00089021	Cox
glucoerucin, glucosinolate	0.95 (0.86, 1.05)	0.3199722	0.62148446	0.0009315	Cox
glucoerysolin, glucosinolate	1.01 (0.93, 1.10)	0.76254108	0.90607823	0.00094395	Cox
glucoibervirin, glucosinolate	0.97 (0.89, 1.07)	0.58967114	0.79409046	0.00088511	Cox
gluconapin, glucosinolate	1.01 (0.94, 1.07)	0.83777279	0.94519611	0.00092354	Cox
gluconasturtiin, glucosinolate	0.92 (0.79, 1.06)	0.25302966	0.57576689	0.0007227	Cox
glucoraphenin, glucosinolate	0.96 (0.87, 1.06)	0.45455642	0.71582247	0.00094096	Cox
choline from glycerophosphocholine	1.17 (1.08, 1.26)	3.7144E-05	0.00184295	0.00044745	Cox
hydroxyproline	0.96 (0.88, 1.05)	0.33757253	0.63138565	0.00088068	Cox
iron	1.01 (0.92, 1.10)	0.87692768	0.94519611	0.0009218	Cox
lactose	1.16 (1.08, 1.24)	4.9379E-05	0.00184295	0.00020755	Cox
lutein and zeaxanthin	0.96 (0.87, 1.05)	0.38912121	0.65612053	0.00081078	Cox
magnesium	0.96 (0.88, 1.04)	0.30399136	0.62148446	0.00081075	Cox
manganese	0.86 (0.78, 0.95)	0.0021239	0.02681429	0.00067254	Cox
napoleiferin, glucosinolate	1.02 (0.94, 1.11)	0.60331798	0.80177785	0.00093992	Cox
natural sugar	1.13 (1.04, 1.23)	0.00456809	0.04194336	0.00089614	Cox
niacin	0.95 (0.86, 1.04)	0.27805905	0.60839568	0.00088993	Cox
oxalate, 2006	0.96 (0.87, 1.06)	0.41209484	0.66278503	0.00089592	Cox
pantothenic acid	1.06 (0.98, 1.14)	0.15542675	0.44851718	0.00095967	Cox
choline from phosphocholine	1.10 (1.01, 1.20)	0.02415796	0.13555301	0.0009659	Cox
alpha linolenic acids, sacks, 2007	0.92 (0.84, 1.01)	0.08591796	0.34710857	0.00065565	Cox
gamma linolenic acids, sacks, 2007	0.95 (0.86, 1.04)	0.24803797	0.57576689	0.00081864	Cox
proanthocyanidin, monomers,	0.98 (0.89, 1.08)	0.65838726	0.83121392	0.00035969	Cox
proanthocyanidin, polymers	0.87 (0.79, 0.97)	0.01014888	0.06833578	0.00057102	Cox
sacharin	1.01 (0.93, 1.10)	0.76133255	0.90607823	0.00054068	Cox
sodium	0.96 (0.87, 1.05)	0.35246714	0.64725784	0.0007651	Cox
sucrose	1.08 (1.00, 1.17)	0.05196743	0.23857773	0.00076556	Cox
sucralose sweetener	0.96 (0.87, 1.06)	0.4667607	0.71582247	0.00079143	Cox
total sugars	1.13 (1.04, 1.24)	0.0054455	0.04230732	0.00062272	Cox
palmitelaidic trans fatty acids, sacks, 2007	1.07 (0.98, 1.17)	0.13748459	0.43143395	0.00070249	Cox
total trans oleic fatty acids, sacks, 2007	0.99 (0.91, 1.08)	0.85642988	0.94519611	0.00084046	Cox
taurine	1.03 (0.95, 1.12)	0.47925303	0.72142514	0.00096213	Cox
theobromine	1.01 (0.93, 1.09)	0.88221913	0.94519611	0.00088759	Cox
total flavones	0.97 (0.89, 1.06)	0.5098003	0.73470323	0.00029725	Cox
total flavonols	0.88 (0.79, 0.98)	0.01920017	0.1158162	0.0008233	Cox
apigenin, flavone	0.93 (0.83, 1.05)	0.2565298	0.57576689	0.00019173	Cox
beta tocotrienol	0.84 (0.76, 0.94)	0.00120773	0.01742583	0.00082784	Cox
beta tocopherol	0.83 (0.75, 0.91)	0.00010173	0.00256858	0.00060363	Cox
catechin, flavan-3-ol	1.00 (0.91, 1.10)	0.98637331	0.98637331	0.00085539	Cox
cyanidin, anthocyanidin	0.92 (0.82, 1.02)	0.0988448	0.38397404	0.00077053	Cox
delphinidin, anthocyanidin	1.00 (0.92, 1.10)	0.93536608	0.96399973	0.00039876	Cox
delta tocotrienol	0.96 (0.88, 1.05)	0.38977457	0.65612053	0.00037139	Cox

delta tocopherol	0.94 (0.86, 1.02)	0.1518906	0.44851718	0.00064239	Cox
epicatechin, flavan-3-ol	0.99 (0.90, 1.09)	0.78444361	0.92126516	0.00057128	Cox
eriodictyol, flavonone	0.88 (0.78, 0.99)	0.02765474	0.14700677	0.00033295	Cox
gamma tocotrienol	0.93 (0.86, 1.02)	0.1269416	0.43143395	0.00067357	Cox
isorhamnetin, flavonol	0.82 (0.73, 0.91)	0.00028357	0.00572819	0.00012817	Cox
kaempferol, flavonol	0.98 (0.88, 1.08)	0.61591568	0.8078894	0.00069684	Cox
malvidin, anthocyanidin	0.94 (0.84, 1.06)	0.31656505	0.62148446	0.00043474	Cox
naringenin, flavanone	1.06 (0.98, 1.13)	0.14096357	0.43143395	0.00018665	Cox
pelargonidin, anthocyanidin	0.92 (0.83, 1.02)	0.11002521	0.39687666	0.00081071	Cox
peonidin, anthocyanidin	0.92 (0.81, 1.05)	0.21337504	0.55258664	0.00056216	Cox
petunidin, anthocyanidin	0.92 (0.82, 1.03)	0.13751284	0.43143395	0.00063702	Cox
theaflavin 3'-gallate, flavan-3-ol	0.96 (0.87, 1.07)	0.46776518	0.71582247	0.00068257	Cox
vitamin d	1.11 (1.03, 1.20)	0.00400869	0.04048776	0.00088238	Cox
vegetable protein	0.84 (0.74, 0.96)	0.00891965	0.06434888	0.00074767	Cox
zinc	1.04 (0.95, 1.13)	0.41342036	0.66278503	0.00091966	Cox
lycopene	0.93 (0.84, 1.02)	0.13747421	0.43143395	2.6269E-05	Weibull
maltose	1.04 (0.95, 1.13)	0.40046637	0.66278503	9.4254E-05	Weibull
hesperetin, flavanone	1.04 (0.96, 1.13)	0.36366084	0.65588829	7.5345E-05	Weibull
vitamin c	0.96 (0.88, 1.05)	0.38478598	0.65612053	1.6699E-05	Weibull

Abbreviations: CI, confidence interval; FDR, false discovery rate; HR, hazard ratio.

[a] The energy-adjusted levels of nutrients were estimated as the standardized residuals produced by regressing the nutrient exposure on total energy.

[b] Hazard ratios for dementia with each nutrient were separately examined and adjusted for age, sex, race/ethnicity, years of education, dietary calories, marital status, smoking, BMI categories, physical activity, comorbidity, and disability.

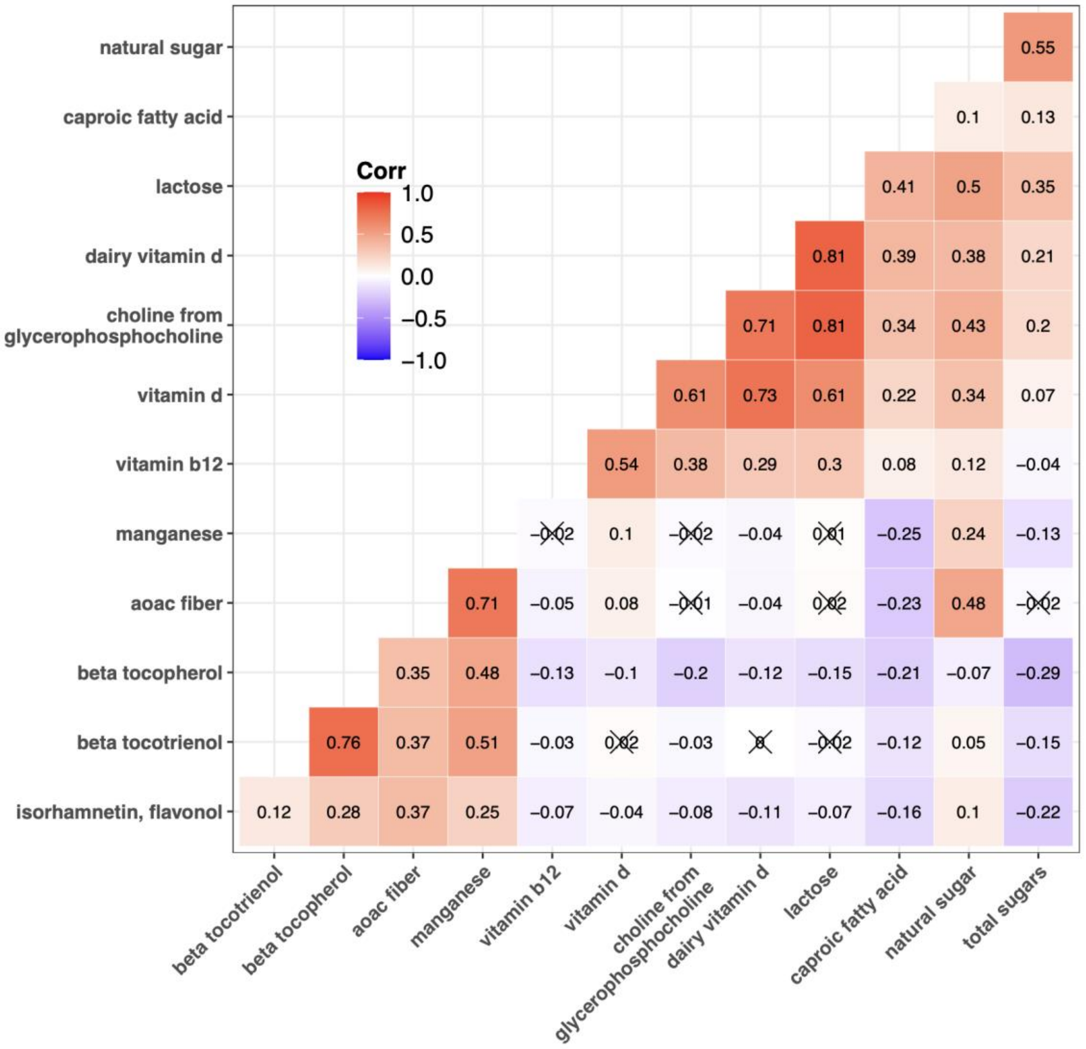

Figure S2. Correlations between selected nutrients. Spearman rank correlations were estimated among thirteen nutrients with FDR-adjusted P values < 0.05 selected from the single-nutrient analysis. Correlation coefficients are shown as texts in the boxes. Darker colors in the boxes mean stronger correlations. All paired correlations were statistically significant ($P < 0.05$) except those marked with cross symbols.

Table S5. Coefficients of selected nutrients based on the elastic net penalized Cox regression model.

Nutrients	Coefficients [a]
natural sugar	0.072370744
vitamin b12	0.065469812
caproic fatty acid	0.056217603
choline from glycerophosphocholine	0.023028422
total sugars	0.013810532
lactose	0.012533641
manganese	-0.016781089
aoac fiber	-0.029063284
beta tocopherol	-0.040225431
beta tocotrienol	-0.05365447
isorhamnetin, flavonol	-0.093124369
dairy vitamin D	0
vitamin D	0

[a] The elastic net penalized Cox regression model was applied to calculate the composite score for 13 nutrients which had FDR-adjusted *P* values < 0.05 in the single-nutrient analysis.

Table S6. Associations between CNS and cognitive decline with mixed-effects linear regression model.

	Model 1 [a]		Model 2 [b]	
CNS	β (95% CI)	*P* value	β (95% CI)	*P* value
Baseline				
CNS categories [c]				
T1 (n = 2094)	Reference		Reference	
T2 (n = 2093)	-0.200 (-0.643, 0.243)	0.377	-0.097 (-0.530, 0.337)	0.662
T3 (n = 2093)	-0.696 (-1.155, -0.237)	0.003	-0.532 (-0.983, -0.080)	0.021
Continuous CNS	-1.127 (-1.971, -0.284)	0.009	-0.798 (-1.633, 0.037)	0.062
Longitudinal				
CNS categories [c]				
T1 × time	Reference		Reference	
T2 × time	-0.018 (-0.082, 0.047)	0.593	-0.016 (-0.080, 0.048)	0.632
T3 × time	-0.078 (-0.144, -0.013)	0.019	-0.072 (-0.137, -0.007)	0.031
Continuous CNS × time	-0.164 (-0.285, -0.043)	0.008	-0.161 (-0.281, -0.040)	0.009

Abbreviations: CNS, composite nutrient score; T1-T3, the 1st to the 3rd tertiles; β, coefficients.

[a] Model 1 was adjusted for age, sex, race/ethnicity, years of education, and dietary calories.

[b] Model 2 was adjusted for covariate in Model 1 plus marital status, smoking, BMI categories, physical activity, comorbidity, and disability.

[c] CNS was categorized into three groups of T1 (≥ -1.390 to ≤ -0.074), T2 (> -0.074 to ≤ 0.072), and T3 (> 0.072 to ≤ 1.44) according to tertiles.

Table S7. Associations between CNS and risks of dementia when additionally adjusting for the status of *APOE* ε4 alleles.

Subpopulation	No. of events/ person-years	HR (95% CI) [a]	*P* value
All (n = 5285)	436/35972	3.36 (2.30, 4.91)	< 0.001
Males (n = 2153)	175/14467	2.08 (1.09, 3.95)	0.026
Females (n = 3132)	261/21505	4.44 (2.78, 7.09)	< 0.001

Abbreviations: *APOE*, apolipoprotein E; CI, confidence interval; CNS, composite nutrient score; HR, hazard ratio.

[a] Cox proportional hazards regression models were applied and adjusted for age, sex, race/ethnicity, years of education, dietary calories, marital status, smoking, BMI categories, physical activity, comorbidity, disability, and status of *APOE* ε4 alleles (with and without ε4 alleles).

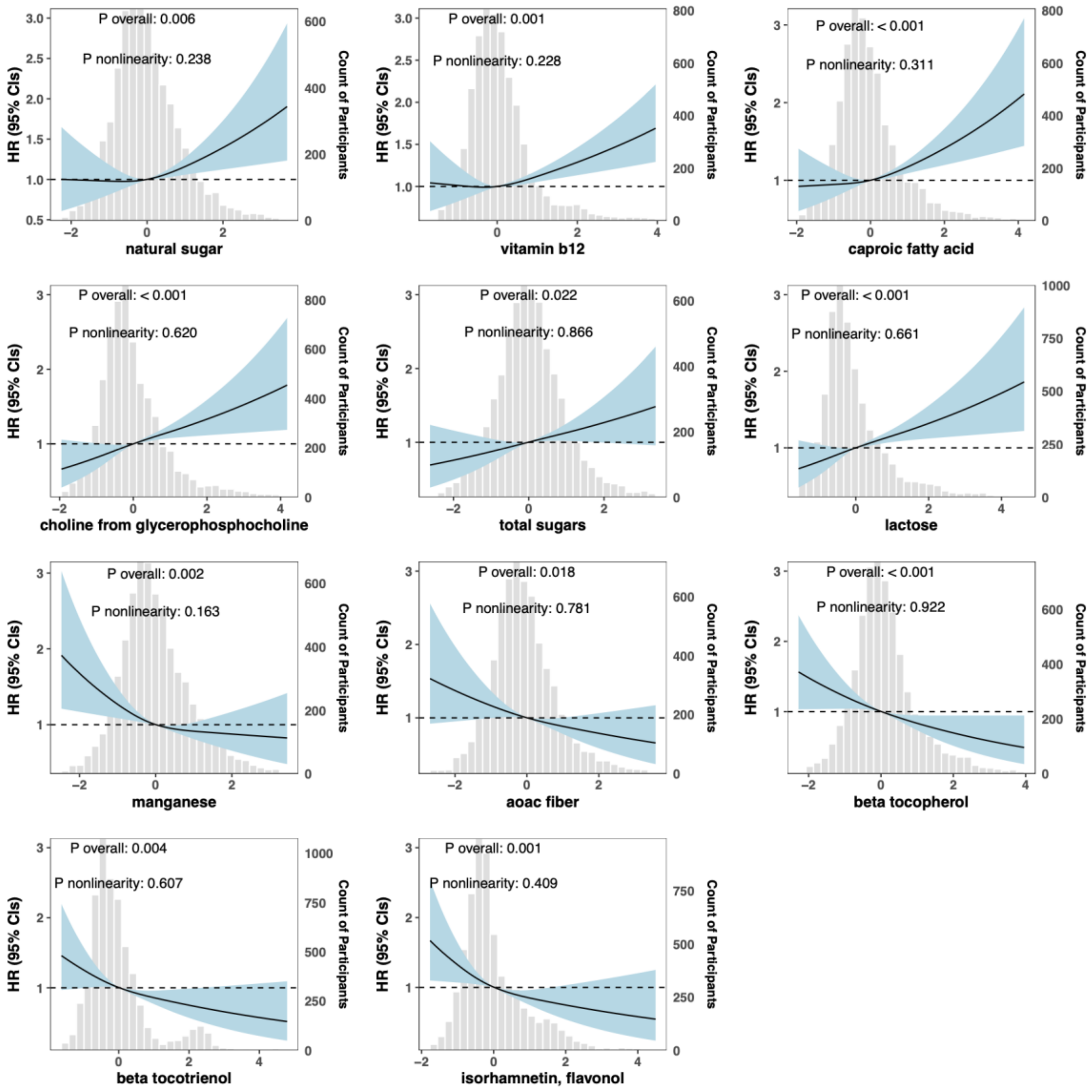

Figure S3. Dose-response associations between selected nutrients and dementia risks. The energy-adjusted levels of nutrients were estimated as the standardized residuals produced by regressing the nutrients on total energy. Eleven nutrients retained in the ENET model were separately analyzed. Restricted cubic splines with three knots at the 10th, 50th, and 90th percentiles of nutrients were included in the fully adjusted Cox regression models to estimate the nutrient-dementia associations. Histograms at bottom show the distributions of nutrients. Abbreviation: CI, confidence interval; ENET, elastic net; HR, hazard ratio.
